# Allele-specific transcription factor binding to common and rare variants associated with disease and gene expression

**DOI:** 10.1007/s00439-016-1654-x

**Published:** 2016-03-18

**Authors:** Marco Cavalli, Gang Pan, Helena Nord, Ola Wallerman, Emelie Wallén Arzt, Olof Berggren, Ingegerd Elvers, Maija-Leena Eloranta, Lars Rönnblom, Kerstin Lindblad Toh, Claes Wadelius

**Affiliations:** 1 Science for Life Laboratory, Department of Immunology, Genetics and Pathology, Uppsala University, Uppsala, Sweden; 2 Science for Life Laboratory, Department of Medical Biochemistry and Microbiology, Uppsala University, Uppsala, Sweden; 3 Department of Biosciences and Nutrition, Center for Biosciences, Karolinska Institute, Novum, Huddinge, Sweden; 4 Science for Life Laboratory, Department of Medical Sciences, Uppsala University, Uppsala, Sweden; 5 Broad Institute of MIT and Harvard, Cambridge, MA USA

**Keywords:** Psoriasis, Primary Biliary Cirrhosis, Rare Variant, Single Nucleotide Polymorphism, High Linkage Disequilibrium

## Abstract

Genome-wide association studies (GWAS) have identified a large number of disease-associated SNPs, but in few cases the functional variant and the gene it controls have been identified. To systematically identify candidate regulatory variants, we sequenced ENCODE cell lines and used public ChIP-seq data to look for transcription factors binding preferentially to one allele. We found 9962 candidate regulatory SNPs, of which 16 % were rare and showed evidence of larger functional effect than common ones. Functionally rare variants may explain divergent GWAS results between populations and are candidates for a partial explanation of the missing heritability. The majority of allele-specific variants (96 %) were specific to a cell type. Furthermore, by examining GWAS loci we found >400 allele-specific candidate SNPs, 141 of which were highly relevant in our cell types. Functionally validated SNPs support identification of an SNP in *SYNGR1* which may expose to the risk of rheumatoid arthritis and primary biliary cirrhosis, as well as an SNP in the last intron of *COG6* exposing to the risk of psoriasis. We propose that by repeating the ChIP-seq experiments of 20 selected transcription factors in three to ten people, the most common polymorphisms can be interrogated for allele-specific binding. Our strategy may help to remove the current bottleneck in functional annotation of the genome.

**Electronic supplementary material:**

The online version of this article (doi:10.1007/s00439-016-1654-x) contains supplementary material, which is available to authorized users.

## Introduction

To date, more than 15,000 single nucleotide polymorphisms (SNP) and insertion/deletions have been associated with diseases and other phenotypes as summarized in the NIH GWAS catalog (Hindorff et al. 2011). It is often assumed that the genetic variant with the highest association is functional; however, this is usually difficult to prove due to linkage disequilibrium (LD) between SNPs (The 1000 Genomes Project. 2012). Only 15 % of the anticipated functional variants are located in coding regions, and many are believed to act through the regulation of gene expression, which is in agreement with the notion that gene activity is largely genetically controlled (Schadt et al. 2008; Lappalainen et al. 2013). Identifying the SNP with the strongest association to gene expression (eSNP) on a haplotype was proposed as a means to finding the variant driving the association to disease, and consequently the NIH started the Genotype Tissue Expression project (GTEX) to correlate a person’s genotype with gene expression in many tissues. However, eSNPs are also generally in LD with other SNPs leaving the question of direct functionality often unanswered. The Encyclopedia of DNA Elements (ENCODE) project (The ENCODE Project Consortium 2012) was initiated with the aim of finding all functional elements in the genome. Data on chromatin and transcribed genes were generated in cell lines and tissues and, based on these, candidate regulatory elements were suggested. These regulatory elements have been shown to harbor an enrichment of GWAS-SNPs (The ENCODE Project Consortium 2012), but despite all efforts, to our knowledge, the functionality of around 20 SNPs have been described, e.g., the ones regulating *SORT1* (Musunuru et al. 2010), *RFX6* (Huang et al. 2014) and *TOX3* (Cowper-Sal et al. 2012). In these cases, a common feature is that the functional SNP is located in a motif for a transcription factor where the alleles differ in their ability to bind the transcription factor (TF) and thus their capacity to regulate one or more genes. After our initial discovery that signals in DNA enriched by chromatin immunoprecipitation (ChIP) may differ between alleles (Ameur et al. 2009), the strategy has been applied genome wide using next-generation sequencing (ChIP-seq) data generated by us (Motallebipour et al. 2009; Wallerman et al. 2009) and others (Kasowski et al. 2010; Rozowsky et al. 2011; Reddy et al. 2012). SNPs with allele-specific (AS) TF binding are likely to be functional and in this project we systematically searched for them. We have characterized AS-SNPs in four major ENCODE cell lines and made functional validations. This has resulted in a collection of 9962 candidate functional SNPs.

## Materials and methods

### Genome sequencing

The genomes of K562 and SK-N-SH were sequenced with paired-end reads to 30× coverage. Illumina sequenced reads were aligned to human reference genome hg19 and duplicates were marked with Picard. The reads then went through GATK preprocessing including indel realignment (DePristo et al. 2011). Variants were called following GATK Best Practices (https://www.broadinstitute.org/gatk/guide/best-practices) using HaplotypeCaller, followed by GVCF file combination and recalibration of variants.

### ChIP-seq sequences

Raw ChiP-seq reads (.fastq) were obtained from the ENCODE project database (ftp://hgdownload.cse.ucsc.edu/goldenPath/hg19/encodeDCC/) selecting all the TF ChIP-seq data available at the time of download for four different cell lines: GM12878 (Nov. 2013), H1-hESC (Jan 2014), K562 (Mar 2014) and SK-N-SH (Mar 2014).

### Genomic features

AS-SNPs collections were intersected and filtered with several publicly available databases: NHGRI GWAS catalog (Jan 2014), collection of signal artifact blacklisted ENCODE regions (The ENCODE Project Consortium 2012), 1000 Genomes SNPs collection (1000 Genomes project, phase1_release_v3.20101123) and lymphoblastoid cell lines eQTLs collections (Lappalainen et al. 2013).

The reference genome (G1) used was the UCSC hg19 assembly based on the Genome Reference Consortium Human genome build 37 (GRCh37), but excluding random and unplaced contigs. The alternative genomes (G2), when not available, were built for the different cell lines using the FastaAlternateReferenceMaker GATK utility that generates an alternative reference sequence replacing the reference bases at variation sites with the bases supplied by a cell-specific SNPs collection. The genomes of K562 and SK-N-SH were sequenced at the Broad Institute as part of this project and the sources for cell-specific SNPs collections and alternative genomes were: 1. GM12878: SNPs from 1000 Genomes pilot project official release and HiSeq from Broad and diploid sequence of NA12878 genome from M.Gerstein laboratory. 2. H1-hESC: SNPs calls were obtained from B.Ren laboratory (January 2014) and G2 built via GATK. 3. K562: WGS and SNP calls from Broad Institute (June 2014) and G2 built via GATK. 4. SK-N-SH: WGS and SNP calls from Broad Institute (June 2014) and G2 built via GATK.

### AS-SNPs selection pipeline

A graphical overview of the AS-SNPs selection process is presented in the flowchart in Fig. S1 in Supplementary material 1. The pipeline follows these steps: (1) each sets of ChIP-seq reads is aligned to the reference (G1) and alternative (G2) genome using ASAP (http://www.bioinformatics.babraham.ac.uk/projects/ASAP/). ASAP uses bowtie with the following flags: best-l 35-n 1, returning hits with a maximum number of one mismatch permitted in the seed (in this case 35 bp). (2) Reads mapped specifically to G1 or G2 are counted at the heterozygous SNPs. SNPs with “0” reads mapped on G1 or G2 are discarded. (3) To determine whether the G1/G2 read counts difference is statistically significant, a binomial test is applied against the null hypothesis of an equal G1:G2 coverage. After correcting for multiple testing (Benjamini & Hochberg or FDR), AS-SNPs with *P* < 0.05 are selected. (4) AS-SNPs are then intersected with the whole 1000 Genomes SNPs collection to retrieve AFs. AS-SNPs with AF ≥1 % are considered common; AS-SNPs with AF <1 % are considered rare as well as AS-SNPs not present in the 1000 Genomes SNPs collection. (5) Extensive filtering of the selected AS-SNPs is performed to remove AS-SNPs that are in centromeric or telomeric regions (UCSC Gap table ±1 Mb), blacklisted ENCODE regions (±100 bp) or not genotyped CNVs. If the AS-SNPs are in CNVs with established allelic copy numbers, they are re-evaluated for statistical significance with adjusted probabilities in the binomial test. Collections of annotated CNV were obtained from literature and publicly available databases:

- H1—CNVs defined in Närvä et al. (2010) (not haplotype resolved).
- GM12878—ENCODE annotated CNVs via Illumina’s Human 1M-Duo Infinium HD BeadChip assay and CNV analysis by circular binary segmentation (CBS) (not haplotype resolved).
- SK-N-SH—CNVs defined from Nexus Copy Number (BioDiscovery) analysis of SNP array data (GSM888680) using SNPRank segmentation algorithm with default settings followed by TAPS (Mayrhofer et al. 2013) (haplotype resolved).
- K562—CNVs defined from Nexus Copy Number (BioDiscovery) analysis of SNP array data (GSM888266) using SNPRank segmentation algorithm with default settings followed by TAPS (Mayrhofer et al. 2013) (haplotype resolved).

In the cases where haplotype-resolved CNVs were available, the null hypothesis in the binomial tests to determine a statistically significant difference in reads count at different alleles (*H*_0_ *: p*_1_ = *p*_2_ = 1/2) was adjusted to take into account the extra chromosomal copies, e.g., if a region was defined as a three-copy CNV and no haplotype info was available, the AS-SNPs overlapping this region were discarded. If haplotype info was available for the same region (namely, which allele was present in a 2:1 ratio), the AS-SNPs overlapping the region were tested against an adjusted null hypothesis (*H*_0_ *: p*_1_ = 1/3|*p*_*2*_ = 2/3), so as to exclude the copy number bias from the significance of the allele-specific binding difference. Regions with four or more copies were excluded from analysis. (6) Pruned AS-SNPs selections are finally intersected with collections of GWAS or eSNPs and SNPs in LD (*r* ^2^ > 0.8) with GWAS or eSNPs (proxy SNPs calculated via the SNAP tool (Johnson et al. 2008) which uses genotype data from the International HapMap Project) to select candidate functional AS-SNPs for experimental validation. We compared the ratio between the allele with higher read number count over the total read count at hz SNPs [G1 if G1 > G2|G2 if G2 > G1/(G1 + G2)] to estimate the allele-specific effect of an SNP. *P* values comparing AS effects between common and rare AS-SNPs were calculated using two-tailed *t* test.

### Cell cultures

Peripheral blood mononuclear cells (PBMCs) were purified from healthy donor buffy coats (Department of Transfusion Medicine, Uppsala University Hospital, Sweden) using Ficoll density-gradient centrifugation. The B cells were isolated from PBMCs by positive selection using CD19+ B-cell isolation kit (Miltenyi Biotec) according to manufacturer’s instructions. The cells were cultured in 1 ml volumes in 24-well plates (Nunc) in macrophage serum-free medium (Life Technologies) at the concentration of 3 × 10^6^ B cells/ml. The cells were stimulated with a phosphorothioate-modified CpG A oligonucleotide ODN2216 (CyberGene) at the concentration of 3 μg/ml and incubated for 5 h at 37 °C with 5 % CO_2_. The harvested cells were stored in RLT buffer (QIAGEN) at −80 °C.

### Gene expression studies

Total RNA was extracted from mock and stimulated B cells using RNeasy^®^ Mini Kit (QIAGEN). First-strand cDNA was synthesized from 1 ng of total RNA with oligo (dT) primer using the Maxima First Strand cDNA synthesis kit (Thermo Scientific). The reaction was performed at 50 °C for 30 min and terminated at 85 °C for 5 min. qPCR gene expression quantifications were performed in 96-well plates using the StepOnePlus™ Real-Time PCR System (Applied Biosystems). Reactions were performed in a total 20 µl volume comprising 10 µl JumpStart™ Taq ReadyMix™ (Sigma-Aldrich) + EvaGreen^®^ dye 20× (Biotium) and 0.25 µg of each primer. All qPCR reactions were performed in triplicate and the Ct values were averaged. Normalization was performed using the housekeeping gene β-ACTIN to evaluate the relative expression.

## Results

### Sequence and allele-specific signals in ENCODE cell lines

We set out to discover putative functional genetic variants by detecting SNPs with a difference in TF binding between alleles at a large scale. We sequenced the genomes of cell lines from myeloid cells (K562) and neuroblastoma (SK-N-SH) (see “Materials and methods”) and downloaded public data on the genome sequence of B cell (GM12878) and embryonic stem cell (H1-hESC). All public ChIP-seq data from the ENCODE project from these cell lines were downloaded, giving data from on average 66 TFs per cell line (range 27–100). We used the allele-specific alignment pipeline (ASAP) (http://www.bioinformatics.babraham.ac.uk/projects/ASAP/) to align the reads to the reference (G1) and alternative (G2) alleles, respectively. We required that at least one ChIP-seq read mapped to each allele to further support the SNP calling. The number of reads mapping to the G1 and G2 allele was counted at all heterozygous positions, and those with a statistically significant difference in the number of reads were identified after correcting for multiple testing and copy number variation (CNV) (see “Materials and methods”). Data from the 1000 Genomes project (The 1000 Genomes Project 2012) was used to determine if the variants were common with allele frequency (AF) ≥1 % or rare <1 %. We used extensive filtering to remove potential false positives in repeated and ENCODE “blacklisted” sequences. The number of reads mapping to the two alternative genomes is presented in Table S1 in Supplementary material 1. There were only small differences in the number of the aligned reads between the genomes, as in previous studies (Rozowsky et al. 2011), indicating that aligning bias toward the reference genome was well controlled for.

In the four cell lines, we found evidence of allele-specific binding of transcription factors to 9962 SNPs (AS-SNPs). The highest number of AS-SNPs, 4299, was detected in K562 and the lowest number, 1014, in H1-hESC (Table 1), and as expected cells with the highest number of data sets of ChIP-seq of TFs had the highest number of AS-SNPs. Annotation in ChromHMM was available for GM12878, K562 and H1-hESC and 17 % of the AS-SNPs were located in active promoters, 17 % in insulators and the rest in distal regulatory elements. Since we only consider heterozygous positions in this analysis, we compared the number of heterozygous SNPs and the number of AS-SNPs that were shared between two or more cell lines. Out of the 5,523,883 heterozygous SNPs, we found 3,296,857 that were unique to one of the cells, whereas 1,580,106, 551,933 and 94,987 were present in 2, 3 and 4 cell lines (Table S2 in Supplementary material 1). The majority of the AS-SNPs, 9215, were detected in only one cell line, while 324, 29 and 3 were shared between 2, 3 and 4 cell lines, respectively. There was a highly significant difference in the distribution of heterozygous and AS-SNPs in these cells (*P* < 2 × 10^−16^), suggesting that most functional gene-regulatory elements are unique to a cell type and that only a small fraction is shared between two or more cell types. Out of our AS-SNPs, 1191 were also detected as DHS variants (Maurano et al. 2015). The results from both strategies depend on the alleles that are present in the studied cell types, so it is expected that the overlap will only be partial.

**Table 1:** AS-SNPs detected in four different cell lines

	Total SNPs called from WGS	Heterozygous SNPs	TFs ChIP-seq-ed	AS-SNPs (total)	Common AS-SNPs (% of total)	Rare AS-SNPs (% of total)
GM12878	3,657,092	2,250,735	86	3265	2631 (81)	634 (19)
H1-hESC	3,377,147	2,014,114	51	1014	858 (85)	156 (15)
SK-N-SH	4,625,100	2,363,834	27	1384	1158 (84)	226 (16)
K562	4,374,809	1,864,966	100	4299	3752 (87)	547 (13)
Total				9962	8399 (84)	1563 (16)

### Many AS-SNPs have low allele frequencies

**Fig. 1:**
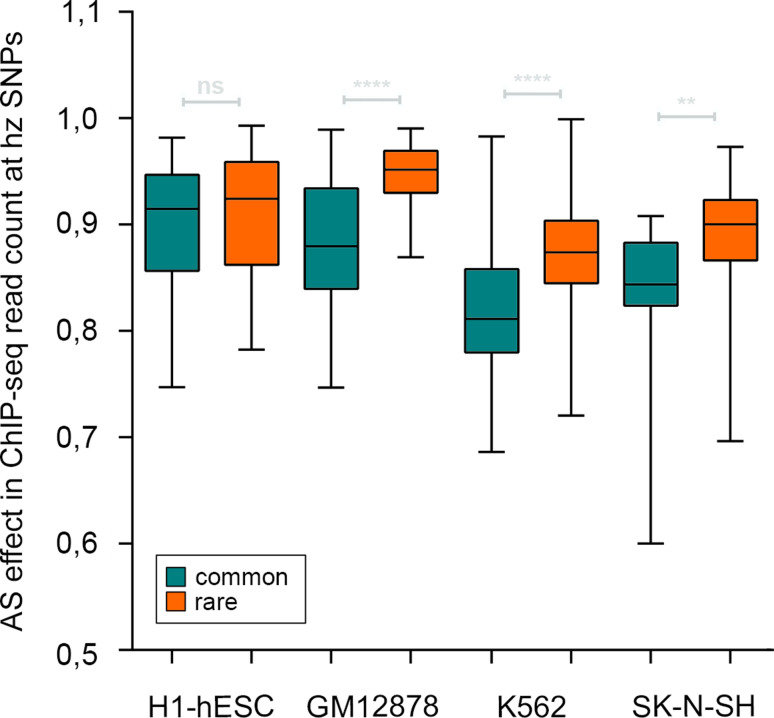
Allelic effects of rare variants. Allele-specific effect (see “Materials and methods”) at heterozygous SNPs with a significant difference between the ChIP-seq reads of alleles G1 and G2 in four different cell lines

Most AS-SNPs are common in the population, but notably 16 % (13–19 %) of all AS-SNPs (1563 AS-SNPs) had an AF <1 %. Out of all heterozygous SNPs in a cell, 14 % have AF <1 % so an equal fraction shows allele-specific TF binding. To estimate the AS effect, we calculated the ratio between the allele with higher read number count over the total read count observed at common or rare heterozygous SNPs (see “Materials and methods”). We found a strikingly higher ratio with rare AS-SNPs in all cells except H1-hESC (Fig. 1), indicating that rare variants may have a larger effect on regulatory elements than common variants. With the exception of H1-hESC, there was no difference in the ratio for AF 1–10 % or 1–5 % compared to common alleles (Fig S5 in Supplementary material 1). Our data suggest that rare variants frequently affect the function of regulatory sequences and their effect may be larger than common alleles (Lappalainen et al. 2013) and that they therefore may contribute to common diseases to a higher degree than rare variants in coding sequences.

### ChIP-seq of 20 TFs, polymerases or coactivators in few unrelated people detect a large fraction of common AS-SNPs in the population

We wanted to determine how many TFs were needed to detect most AS-SNPs in a cell and therefore investigated the fraction identified by the 20 TFs that showed the highest number of allele-specific binding sites in each cell line. We found that between 92 % and 99 % of all AS-SNPs in each cell line were detected by the top 20 TFs, polymerases or coactivators, and even if ChIP-sequencing of 100 different DNA-binding proteins had been performed, the results from the top 20 are enough to detect most AS effects in a cell. In a project to search B cells, or the other cells studied here, from additional people for AS-SNPs, these TFs can be selected to optimize the chances of finding candidate regulatory variants.

**Fig. 2:**
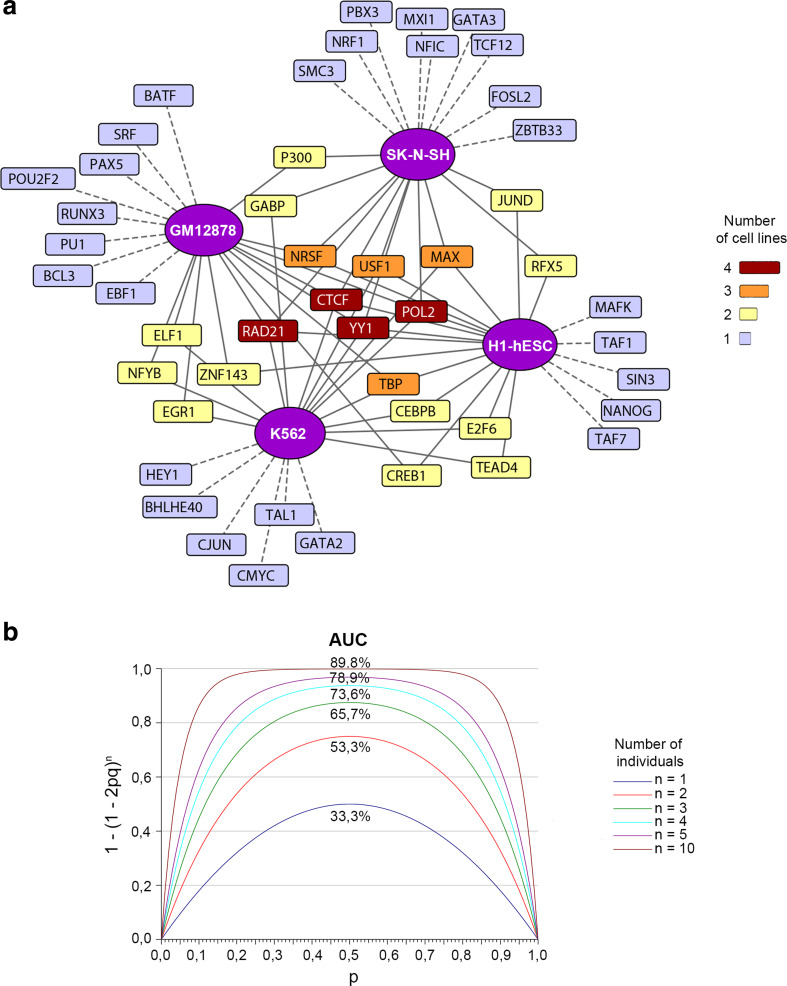
Coverage of transcription factors and alleles in the population. **a** Network representing the top 20 TFs, polymerases or coactivators whose ChIP-seq reads detect most AS-SNPs in four different cell lines. The TFs detecting most AS-SNPs in several cell lines are clustered at the center with the more cell-specific ones in the outer layers. **b** The likelihood of finding a heterozygous SNP as a function of the allele frequency considering one or more individuals. The AUC represents the proportion of heterozygous SNPs a population of *n* individuals

We investigated the overlap of the top 20 TFs, polymerases or coactivators between the four different cell lines (Fig. 2a; Table S4 in Supplementary material 1). Three TFs (CTCF, RAD21, YY1) and POL2 detected many AS-SNPs in all cell lines, three lines shared 4 TFs (NRSF, MAX, USF1 and TBP) and two lines shared 11 TFs (CEBPB, ZNF143, GABP, JUND, RFX5, EGR1, ELF1, NFYB, CREB1, TEAD4 and E2F6) and P300. The number of highly informative TFs that are unique to one cell line varied between five (H1-hESC) and nine (SK-N-SH). Some of these unique TFs are previously known to be of central importance for the cell, i.e., pioneer TFs like NANOG in H1-hESC and factors important for maturation of B cells such as BCL3, EBF1 and PU1 in GM12878. Our data suggest that TFs shared by many cells, pioneer TFs and those important for cell development should be selected when designing a project to identify AS-SNPs in a cell or tissue previously not studied by ChIP-seq.

We then investigated the number of individuals that need to be analyzed to detect most common AS-SNPs in a certain cell type. Assuming Hardy–Weinberg equilibrium (HWE), 33 % (1/3) of all common polymorphisms are heterozygous in one person. There is a huge number of rare variants in the human population and only a small fraction of them are heterozygous in one person. We determined the percentage of common polymorphisms expected to be heterozygous in two to ten unrelated individuals assuming HWE. According to our prediction (Fig. 2b and Table S5), 66 % (2/3) of all common polymorphisms are expected to be heterozygous in at least one of three unrelated people. For five people, the corresponding figure is 79 % and for ten people 90 %. The likelihood to find at least one heterozygous person depends on the AF and is the highest at AF = 0.5. At this AF, you have 88 % chance of discovering a heterozygote if you study three people, 97 % if you study five and over 99 % in ten people. However, even at AF = 0.1, the chance to find one or more heterozygotes is 45 % among three people, 63 % among five and 86 % in ten people. For rare variants (AF <1 %), the chances decrease substantially. It is thus possible to search for AS-SNPs among most common polymorphisms in the population by investigating cell lines or tissues from three to ten people, ideally from diverse ethnic backgrounds.

### Many AS-SNPs are associated with disease and gene expression

**Fig. 3:**
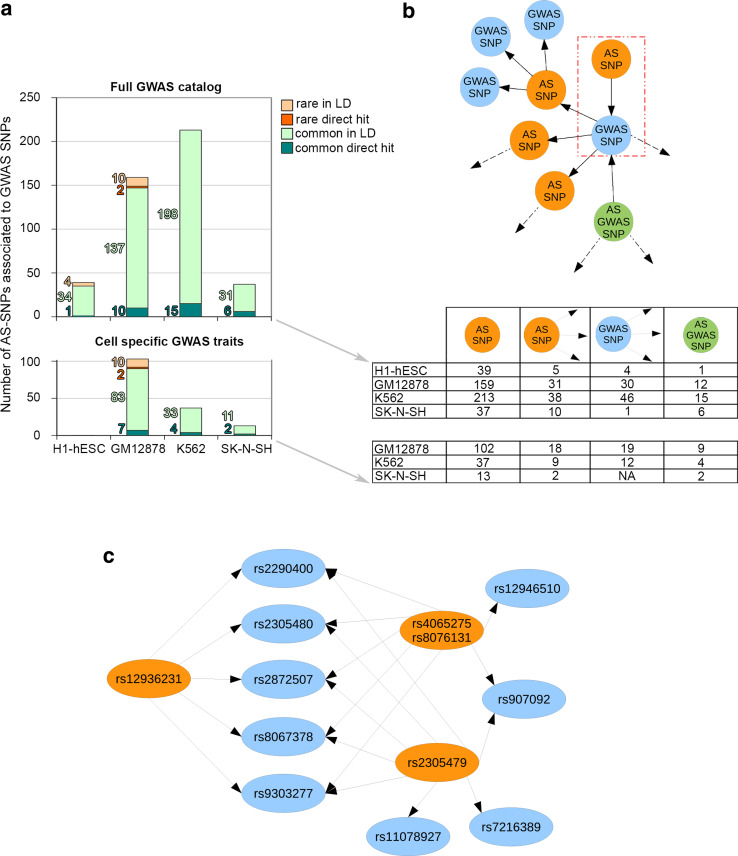
AS-SNPs associated with GWAS-SNPs. **a** *Top* AS-SNPs associated with GWAS-SNPs intersecting the cell-specific collections of AS-SNPs with the full GWAS catalog. Numbers are reported for common and rare AS-SNPs that are direct hits in the GWAS catalog or in LD with GWAS-SNPs. *Bottom* AS-SNPs associated with GWAS-SNPs intersecting the cell-specific collections of AS-SNPs with GWAS-SNPs associated with cell-specific traits. **b** Model representation of the networks of interactions observed between AS-SNPs and GWAS-SNPs. The dotted red box highlights the simplest scenario with one AS-SNP in LD with a GWAS-SNP. The tables report the numbers of instances observed in each cell line where one AS-SNP is in LD with several GWAS-SNPs or one GWAS-SNPs is in LD with different AS-SNPs, or where GWAS-SNPs were also AS-SNPs. **c** Four AS-SNPs, located in three different regulatory elements, interact with several GWAS-SNPs associated with autoimmune diseases

The GWAS catalog has entries for a wide spectrum of traits that are expressed in different cell types, all of which were not studied in this experiment. Molecular events that could be explained by the cells investigated are for example immune-mediated diseases for GM12878 and K562, and neurological diseases for SK-N-SH (Table S3 in Supplementary material 1). Consequently, we searched the catalog for these cell-specific traits. H1-hESC was not investigated. The SNP with the strongest association (GWAS top hit) was collected from the catalog and SNPs in high LD (*r* ^2^ > 0.8) were identified and intersected with the collection of AS-SNPs. For 36 traits, we found 141 AS-SNPs. We investigated the number of candidate regulatory SNPs found when searching the GWAS catalog using matched random sets of non-AS-SNPs. We found significantly more AS-SNPs than expected by chance with a clear enrichment (5- to 13-fold) in the overlap of AS-SNPs as compared to random sets of non-AS-SNPs (Fig S3 in Supplementary material 1). Out of the 141 unique AS-SNPs that were candidates to explain GWAS signals, only 15 were the particular SNP reported in the GWAS catalog and the other 126 were in the high-LD interval and 10 of these were rare AS-SNPs (Table 2; Fig. 3a, b and Tables S8, S11, S13 in Supplementary material 3). We grouped the SNPs in 1 Mb loci, and in immune cells, GM12878 and K562, we found candidate functional SNPs at 71 loci and in SK-N-SH at 14 loci, giving a total of functional candidates at 85 loci. We compared our candidates with the total number of mapped regions for different diseases and detected tentative functional variants for 11 % of loci mapped for psoriasis, 9 % for SLE and self-reported allergy, 8 % for inflammatory bowel disease/Crohn’s disease/ulcerative colitis and 6 % for type 1 diabetes, suggesting new functional regulatory elements for many common diseases.

**Table 2:** AS-SNPs detected in different cell lines associated with cell-specific GWAS traits

GWAS-associated traits	Number of AS-SNPs	Number of AS-loci	Number of reported loci ¤	% of reported loci with AS-SNPs
GM12878 and K562				
Systemic lupus erythematosus	28 (18^a^)	6	63	9, 5
Crohn’s disease|inflammatory bowel disease|ulcerative colitis	22 (3^a^)	14	182	7, 7
Type 1 diabetes	14 (7^a^)	4	65	6, 2
Chronic lymphocytic leukemia	14 (14^a^)	1	25	4, 0
Asthma	13 (8^a^)	3	74	4, 1
IgG glycosylation	11 (1^a^)	9	295	3, 1
Schizophrenia	18	3	101	3, 0
Self-reported allergy	12	3	34	8, 8
Rheumatoid arthritis	9	4	151	2, 6
Psoriasis	6	3	27	11, 1
Systemic sclerosis	5	2	27	7, 4
Celiac disease	5	3	61	4, 9
Primary biliary cirrhosis	4	1	24	4, 2
Adverse response to chemotherapy (neutropenia/leucopenia) (carboplatin)	4	2	9	22, 2
Chronic myeloid leukemia	3	1	28	3, 6
Multiple sclerosis	3	3	138	2, 2
Amyotrophic lateral sclerosis	2	1	142	0, 7
Restless legs syndrome	2	1	6	16, 7
Vitiligo	2	1	33	3, 0
Acute lymphoblastic leukemia (childhood)	1	1	33	3, 0
Ankylosing spondylitis	1	1	21	4, 8
Atopic dermatitis	1	1	18	5, 6
Endometriosis	1	1	20	5, 0
IgE grass sensitization	1	1	9	11, 1
Primary sclerosing cholangitis	1	1	2	50, 0
SK-N-SH				
Obesity-related traits	3	3	572	0, 5
Parkinson’s disease	2	2	50	4, 0
Schizophrenia	1	1	101	1, 0
Bipolar disorder and schizophrenia	1	1	113	0, 9
Attention deficit hyperactivity disorder (time to onset)	1	1	128	0, 8
Alzheimer’s disease (late onset)	1	1	48	2, 1
Inattentive symptoms	1	1	14	7, 1
Amyotrophic lateral sclerosis	1	1	142	0, 7
White matter integrity	1	1	14	7, 1
Anorexia nervosa	1	1	26	3, 8
Migraine	1	1	60	1, 7

Loci defined as GWAS-SNPs within 1 Mb regions

^a^In HLA region

Furthermore, we took the top hits from the whole GWAS catalog including SNPs in high LD (*r* ^2^ > 0.8) and compared to the list of AS-SNPs. This resulted in 398 entries of common and 14 rare AS-SNPs (Tables S9, S12, S14 and S15 in Supplementary material 3). The relevance of these AS-SNPs is not ascertained, since they were not necessarily detected in the appropriate cell to explain the phenotype, but it is likely that several of them may be functional due to pleiotropic effects. We have found novel putative functional variants that may explain GWAS-SNPs using lymphoblastoid cell lines. Some examples are AS-SNPs detected in GM12878 and K562 on a haplotype at the *IKZF3/ORMDL3* locus, which is associated with both a range of autoimmune diseases described below and cervical cancer. Infection with papilloma virus is a strong risk factor for cervical cancer and variation in the immune response to this agent may contribute to disease risk. This reflects that cellular mechanisms may be shared between cells types, and a gene-regulatory element detected in one cell type could be relevant to other types.

We have also found novel putative functional variants that may explain eQTL SNPs (eSNPs). Genetic control of gene activity has been investigated in lymphoblastoid cell lines and eSNPs have been detected (Lappalainen et al. 2013). We examined whether AS-SNPs in B cells (GM12878) are better candidates to drive the allelic difference in expression. We took SNPs in LD (*r* ^2^ > 0.8) with 16,078 eSNPs for B cells and investigated how many are AS-SNPs in GM12878. We found 84 eSNPs that are AS-SNPs, and an additional 362 AS-SNPs that are in LD with an eSNP (Tables S10 in Supplementary material 3). Some of the eSNPs in GM12878 are associated with the expression of repeats and therefore difficult to interpret from a disease perspective. We consider the AS-SNPs as good candidates to drive the allele-specific expression variation and note that only a fraction of eSNPs shows tentative functional effect, but that many more SNPs in high LD do. There is a clear enrichment (5- to 13-fold) in the overlap of AS-SNPs and cell-specific GWAS and eQTLs compared to random sets of non-AS-SNPs (Fig S3 in Supplementary material 1).

### Networks of AS-SNPs and GWAS-SNPs

A haplotype at the *IKZF3/ORMDL3* locus is associated with primary biliary cirrhosis, systemic lupus erythematosus, systemic sclerosis, Type 1 diabetes autoantibodies, type 1 diabetes, ulcerative colitis, Crohn’s disease, inflammatory bowel disease and rheumatoid arthritis (Hindorff et al. 2011) (Fig. 3c and Fig. S2 in Supplementary material 1). Seven SNPs detect the associations, but none are AS-SNPs. However, they are in LD with four AS-SNPs: AS-SNPs rs4065275 and rs8076131 separated by 47 bp at one regulatory element, AS-SNP rs2305479 residing 18 kb away and to AS-SNP rs12936231 (Table S8 in Supplementary material 1) located a further 33 kb away at another element (Verlaan et al. 2009). Our data suggest that the AS-SNPs, all detected in the B cell GM12878, are good candidates to drive the association and that they may influence the expression of *IKZF3* which is important for B lymphocyte proliferation and differentiation or *ORMDL3* involved in sphingolipid synthesis. The pattern that several GWAS-SNPs are associated with the same AS-SNP and that several AS-SNPs are detected at one locus is found in many other instances (Fig. 3b). This suggests that SNPs at distinct regulatory elements could regulate the activity of the same gene, which is supported by recent data (Corradin et al. 2014). To test if AS-SNPs are enriched in super-enhancers we obtained a collection of ~60,000 super-enhancers (dbSUPER, http://bioinfo.au.tsinghua.edu.cn/dbsuper/index.php) defined per cell line and intersected them with our collections of AS-SNPs or random sets of selected non-AS-SNPs (Fig. S4 in Supplementary material 1). We found a higher fraction of super-enhancers to contain AS-SNPs than what would be expected by chance (*P* = 2 × 10^−5^).

### An AS-SNP regulates *SYNGR1,* a candidate for rheumatoid arthritis and primary biliary cirrhosis

**Fig. 4:**
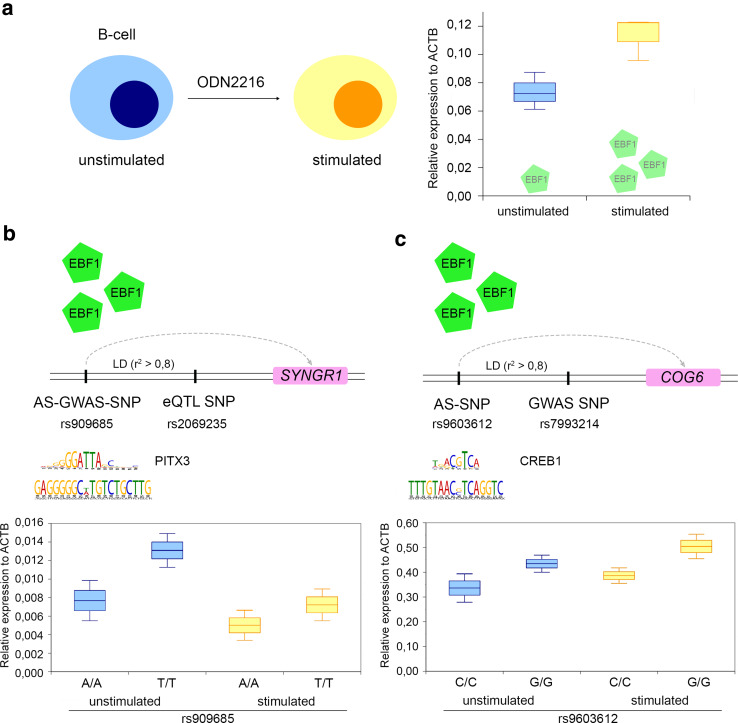
Functional effects of AS-SNPs in primary B cells. **a** Stimulation of B cells with the oligonucleotide ODN2216 increases the expression of TF EBF1. **b** *Top* rs909685 is a GWAS-SNP with AS behavior detected by EBF1 ChIP-seq reads. rs909685 is in LD with the eQTL SNP rs2069235 which is associated with the expression of the *SYNGR1* gene. *Bottom* *SYNGR1* expression in an individual homozygous for the A- or T-allele, respectively, at rs909685, unstimulated (*blue*) or stimulated with ODN2216 (*yellow*). *Middle* motif for PITX3 at rs909685 which alters the TF-binding motif. **c** *Top* AS-SNP rs9603612 is located in an intron of *COG6* and is in LD with the GWAS-SNP rs7993214. rs9603612 showed AS behavior with EBF1 ChIP-seq reads covering the SNP with significant difference. *Bottom* expression of *COG6* in an individual homozygous for the C- and G-alleles, respectively, of rs9603612, unstimulated (*blue*) or stimulated with ODN2216 (*yellow*). *Middle* motif for CREB1 at rs9603612 which alters the TF-binding motif

Recently, rs909685 was associated with rheumatoid (Okada et al. 2014) and primary biliary cirrhosis (Liu et al. 2012). The eSNP rs2069235 is in high LD with the GWAS/AS-SNP rs909685 and associated with the expression of *SYNGR1*, coding for a membrane protein associated with presynaptic vesicles in neuronal cells and also expressed in B- and other immune cells. To validate the results from GM12878, we purified B cells from blood donors who had been genotyped using a 200 K ImmunoChip. The cells were either treated with medium only (mock) or stimulated with the oligonucleotide ODN2216 (see “Materials and methods”), which activated the TF EBF1 (Fig. 4a). ChIP-seq has shown that EBF1 binds to the regulatory element that harbors the GWAS/AS-SNP rs909685. B cells from donors who are homozygous, AA or TT for the alleles, respectively, at this locus showed significant difference in activity of *SYNGR1* (Fig. 4b, lower panel). Stimulation with ODN2216, which increases the levels of EBF1, significantly decreased the expression of *SYNGR1* for both genotypes (Fig. 4b, lower panel). rs909685 disrupts the motif for PITX3 which is known to interact with NURR1, a factor involved in the regulation of cortisol which suggests a link to inflammatory processes (Mages et al. 1994). This indicates that *SYNGR1* is controlled by the regulatory element with the AS-SNP rs909685 giving an allelic difference in expression at basal conditions and that EBF1 acts as a repressor to decrease the expression from both alleles. Furthermore, it suggests that differential expression of *SYNGR1* could mediate the genetic predisposition to rheumatoid arthritis and primary biliary cirrhosis at this locus.

### An AS-SNP regulates psoriasis and rheumatoid arthritis candidate gene *COG6*

A GWAS has shown that rs7993214 is associated with psoriasis (Liu et al. 2008) and rs9603616 to rheumatoid arthritis (Okada et al. 2014). The GWAS-SNPs rs7993214 and rs9603616 are in high LD with each other and with the AS-SNP rs9603612 which is located at a regulatory element bound by EBF1 in GM12878. Purified B cells from blood donors, homozygous, CC or GG, for the alleles at rs9603612 were stimulated with ODN2216 and we determined the expression of the psoriasis candidate gene *COG6*. A donor homozygous for the G allele had significantly higher expression than the one homozygous for C. In the cells stimulated with ODN2216, the expression of *COG6* increased significantly for both genotypes (Fig. 4c, lower panel). This indicates that *COG6* is differentially expressed depending on the allele at rs9603612 and that EBF1 regulates the activity of both alleles. Homozygous mutations in *COG6* cause Shaheen syndrome with hyperkeratosis of the palms and soles, hypohidrosis, intellectual disability and dental enamel hypoplasia. Patients with psoriasis also have hyperkeratosis resulting in scaly patches over the body, in the palms of the hands and soles of the feet. The AS-SNP rs9603612 is located in a motif for CREB1, which is activated in psoriatic lesions (Funding et al. 2007). The data thus suggest that the risk of developing psoriasis is mediated by the regulatory element with the functional SNP rs9603612 leading to differential activity of *COG6*.

## Discussion

Most variants associated with common diseases are in non-coding DNA and enriched in regulatory DNA (Maurano et al. 2012). Many AS variants have been detected using reads from DHS analysis (Maurano et al. 2015) and were predicted to be caused by AS TF binding. As a complementary approach in this study, we present candidate functional SNP variants detected using the biased TF allele binding in ChIP-seq data. ChIP-seq reads generated for TFs in four different cell lines from the ENCODE project were analyzed to identify cell-specific collections of AS-SNPs. This makes a significant addition to the collection of SNPs with AS chromatin signals. One incentive for the study was to define candidate variant drivers of GWAS signals and we identified 141 AS-SNPs meeting the criteria. Based on the detailed validations of two SNPs associated with rheumatoid arthritis/primary biliary cirrhosis and psoriasis, we believe that many others are worth further study. These efforts will make it possible, not only to validate the regulated gene, but also to connect the disease process to upstream regulatory pathways. This will provide a more holistic approach to the understanding of disease processes and help to remove the current bottleneck in the process of translating GWAS signals to functional disease mechanisms.

Most of the detected AS-SNPs are located in distal regulatory elements and are candidates to regulate a nearby gene, while 17 % are located in promoters. Recent studies have indicated the presence of chromatin domains with associated signals between SNPs (Waszak et al. 2015; Grubert et al. 2015), but across the genome, Waszak et al. (2015) detected 14,559 domains, of which only 25 % were larger than 70 kb. Correlation of allelic bias in adjacent SNPs was also found (Maurano et al. 2015) with the same conclusion that most correlations are present in regions with sizes of regulatory elements and only a small fraction extending over 10 kb. This indicates that at a (small) fraction of regions, the AS-SNPs we detect may be correlated to other functional variants which are the drivers of chromatin signals. In B cells, we found 446 AS-SNPs that are eQTL SNPs or are in high LD with such SNPs and we regard them as candidates to drive the eQTL. This does not exclude the possibility that many of the other AS-SNPs may be associated with gene expression variation. A recent study in mice (Crowley et al. 2015) suggests that more than 80 % of mouse genes have cis-regulatory variation. Studies in man have been performed on small sample sets and therefore underpowered to detect the weak effects, so more associations remain to be found as studies increase, e.g., in the GTEx consortium.

Recent data have shown a high number of rare variants in people and the idea is that some of them are functional, and that evolution has not had time to remove them from the population (Keinan and Clark 2012). Exome sequencing has been performed in large disease cohorts and controls and only a low number of rare coding variants have been associated with disease, indicating that they do not have a large impact on disease risk in the population (Fu et al. 2013). We found a high number of candidate functional AS-SNPs that are rare in the population, which can be compared to 53–80 predicted candidate functional variants per person, most of them rare, in the coding sequence (Li et al. 2015; Fu et al. 2013). We have just studied four cell types from one person each, so if all different cells in the human organism would be analyzed, the number of rare candidate-regulatory variants would increase and even more outnumber the rare candidate functional coding variants. We observed a significantly higher difference in G1/G2 read counts at rare AS-SNPs as compared to common ones, which suggests that rare AS-SNPs may have a large functional effect. This is consistent with findings from eQTLs in B cells (Lappalainen et al. 2013), showing that low-frequency alleles have a large effect on expression. It is therefore possible that rare variants in regulatory regions frequently contribute to common disease risk. This possibility has been difficult to study since the correct functional regulatory element needs to be investigated; however, the data we now present points to a collection of candidate regulatory sequences.

If rare variants act on regulatory elements in the frequencies we detect, it would add heterogeneity and noise to association studies. Rare variants are often specific to an ethnic group, and in one population a set of rare variants may be associated with one common variant on a haplotype, whereas in another population there may be one or more rare variants associated with another common SNP. Consequently, different GWAS and eQTL studies may find the strongest signals to different common SNPs on the same haplotype that has one or more common functional variant(s). This is consistent with the fact that GWAS studies often find the strongest association to alternative SNPs and with our finding that several GWAS-SNPs at a locus often show association to one or a few AS-SNPs (Fig. 3). The missing heritability has been much debated over the years. Rare variants are generally not found in GWAS studies and often even filtered out in quality control steps. If rare variants contribute to common diseases in the numbers that we detect them, they may change the proportion of the explained heritability. Not only are they frequent, but their allele-specific effect may be larger than for common ones; so combined, this may explain a part of the missing heritability. We are approaching an era when GWAS studies will be based on whole genome sequencing, thus making it possible to evaluate the contribution of rare regulatory variants to common disease. In the same way, the rare variants may also obscure associations of gene expression and may be the reason why we detect many more AS-SNPs in LD with an eSNP, than AS-SNPs that are eSNPs.

In an attempt to minimize the proportion of false positives during the selection of AS-SNPs, we applied strong filtering and cutoffs to eliminate candidate SNPs in genomic regions with high repetitive content such as centromeres, telomeres and CNVs. In fact, large regions were inaccessible for analysis due to CNVs and large chromosomal abnormalities, limiting the number of AS-SNPs that could be detected. In future projects to find candidate regulatory SNPs, care should be exercised to use cell lines without gross structural rearrangements. The number of detected AS-SNPs depends on the number of reads at each allele and in this data set it was limited by the type of data generated in ENCODE. We propose that a project optimized to search for AS-SNPs should improve power by using long paired-end reads to sequence the whole ChIP-enriched DNA fragment and by generating a high number of reads.

Our results indicate that it is possible to predict which TFs will detect most AS-SNPs in a cell or tissue, which has not been analyzed before. Starting with the TFs that are expressed in the cell or tissue, one can select those that are shared between two or more of the cells in this study and add pioneer factors and others with verified central importance to the cell. As described, ChIP-seq of 20 selected TFs from the cells studied here or 20–30 TFs for new cells or tissues is a powerful way to find a large collection of common AS-SNPs. By repeating the experiment in samples from three to ten people, the most common polymorphisms in the human population can be interrogated for allele-specific binding. This strategy can be used to find more candidate functional variants. Rare variants are, as always, harder to study. They will be detected in whole genome sequencing, but their functional effect may need to be studied by large-scale functional tests (Kheradpour et al. 2013) or by computer modeling.

## Conclusions

Drivers of GWAS signals in non-coding regions are located in gene-regulatory elements and result in difference in TF binding between alleles. We have screened for such events in four cell types and found a large number of candidate functional variants. Those in LD *r* ^2^ > 0.8 in relation to reported SNPs for traits and expression were identified resulting in hundreds of potential genetic drivers. Experimental validation supports SNPs in B cells that explain psoriasis, rheumatoid arthritis and primary biliary cirrhosis. As many as 16 % of functional candidates are rare with AF <1 %. If we assume that they contribute to common disease to the same degree as common variants, they may explain why GWAS signals differ between populations and may contribute to the missing heritability.

## Electronic supplementary material

Below is the link to the electronic supplementary material.

Supplementary material 1 (PDF 1036 kb) **Supplementary material 1**: **Fig. S1**. AS-SNPs discovery flowchart. **Fig. S2**. Genomic context for SNPs interaction network presented in Fig. 3c. **Fig. S3**. Overlap of GM12878 AS-SNPs and non-AS-SNPs with GWAS and eQTL SNPs. **Fig. S4**. Overlap of AS-SNPs and non-AS-SNPs with super-enhancers. **Fig. S5**. Allele specific (AS) effect as a function of allele frequency (AF). **Table S1**. ChIP-seq reads aligned on reference (G1) and alternative (G2) genomes. **Table S2**. Heterozygous and AS-SNPs present in one or several cell lines. **Table S3**. List of cell-specific traits selected form the GWAS catalog. **Table S4**. Top 20 TFs, polymerases or coactivators detecting most AS-SNPs in 4 cell lines. **Table S5.** Fraction of heterozygous SNPs in one or more individuals based on allele frequencies. **Table S6**. Oligonucleotide sequences used in this study

Supplementary material 2 (XLSX 354 kb) **Supplementary material 2**: **Table S7.** Complete collection of AS-SNPs identified in 4 cell lines

Supplementary material 3 (XLSX 109 kb) **Supplementary material 3**: **Tables S8-15.** Cell-specific AS-SNPs collections associate to GWAS and eQTL SNPs
