# Supplementary material for: Allele-specific transcription factor binding to common and rare variants associated with disease and gene expression
Source: Hum Genet. 2016 Mar 18;135(5):485–97. doi: 10.1007/s00439-016-1654-x (PMC4835527; doi:10.1007/s00439-016-1654-x)
Supplement: Supplementary file 1 [file 439_2016_1654_MOESM1_ESM.pdf]

Supplementary Figures and Tables to:

Allele-specific transcription factor binding to common and rare variants associated to disease and gene expression

Marco Cavalli, Gang Pan, Helena Nord, Ola Wallerman, Emelie Wallén Arzt, Olof Berggren, Ingegerd Elvers, Maija-Leena Eloranta, Lars Rönnblom, Kerstin Lindblad Toh, Claes Wadelius

Contents

Fig. S1. AS-SNPs discovery flowchart. 2

Fig. S2. Genomic context for the SNPs interaction network presented in Fig. 3C. 3

Fig. S3. Overlap of GM12878 AS-SNPs and non-AS-SNPs with GWAS and eQTL SNPs. 4

Fig. S4. Overlap of AS-SNPs and non-AS-SNPs with super-enhancers 5

Fig. S5. Allele specific (AS) effect as a function of allele frequency (AF) 6

Table S1. ChIP-seq reads aligned on reference (G1) and alternative (G2) genomes. 7

Table S2. Heterozygous and AS-SNPs present in one or several cell lines. 7

Table S3. List of cell-specific traits selected form the GWAS catalog. 8

Table S4. Top 20 TFs, polymerases or coactivators detecting most AS-SNPs in 4 cell lines. 11

Table S5. Fraction of heterozygous SNPs in one or more individuals based on allele frequencies. 12

Table S6. Oligonucleotide sequences used in this study. 13

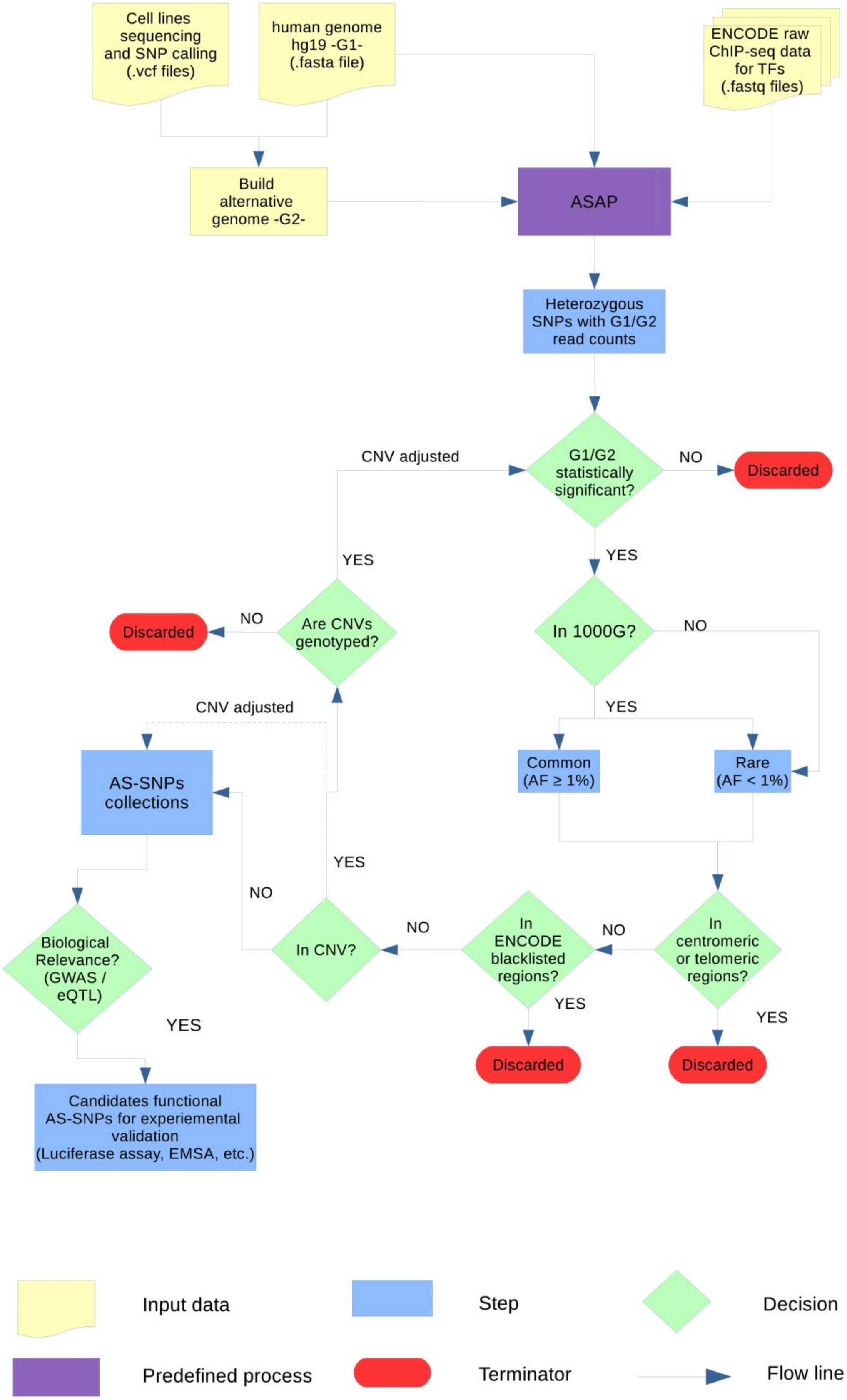

Fig. S1. AS-SNPs discovery flowchart.

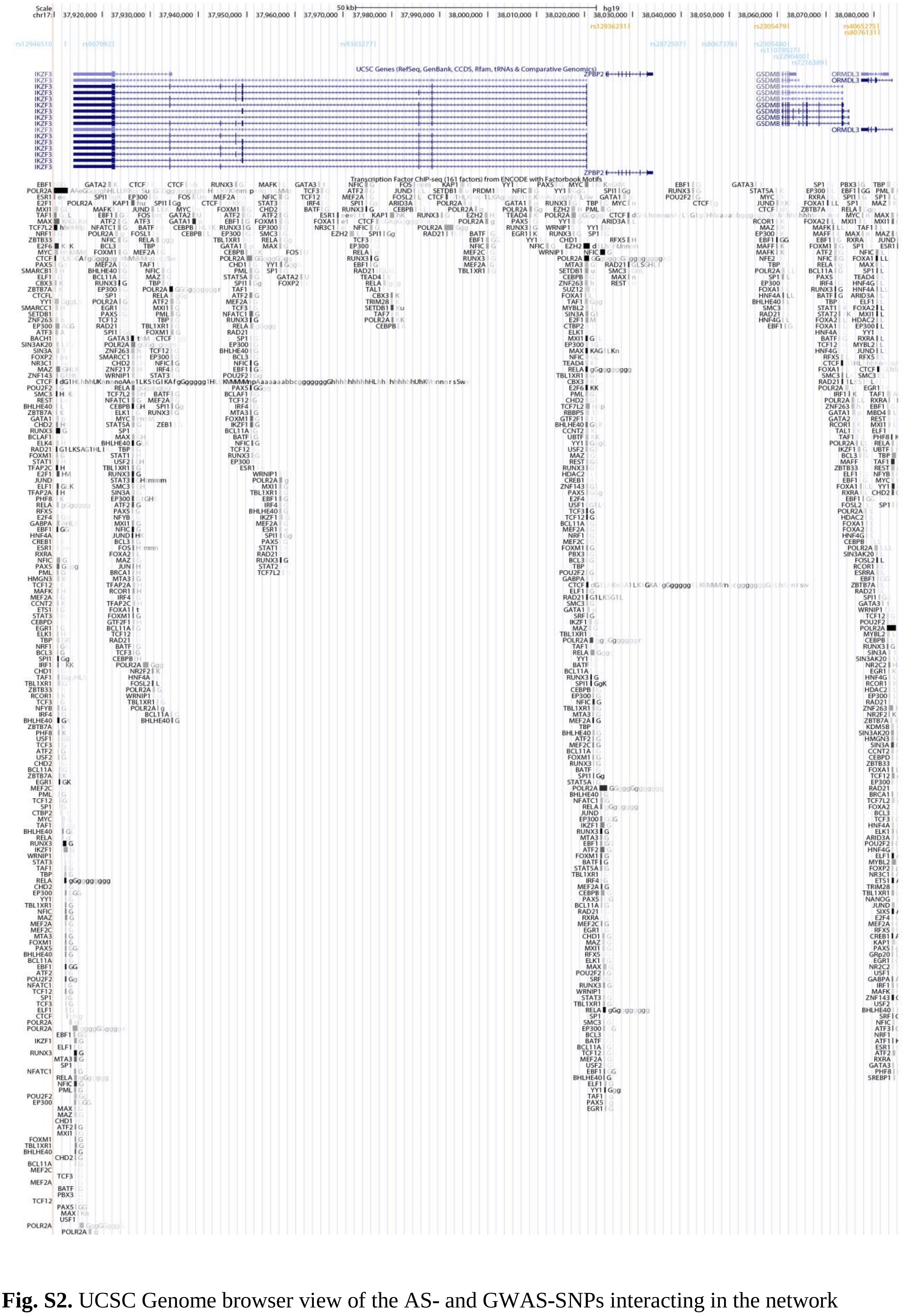

Fig. S2. UCSC Genome browser view of the AS- and GWAS-SNPs interacting in the network presented in Fig. 3C. The SNPs are highlighted with the same color code as per Fig. 3.

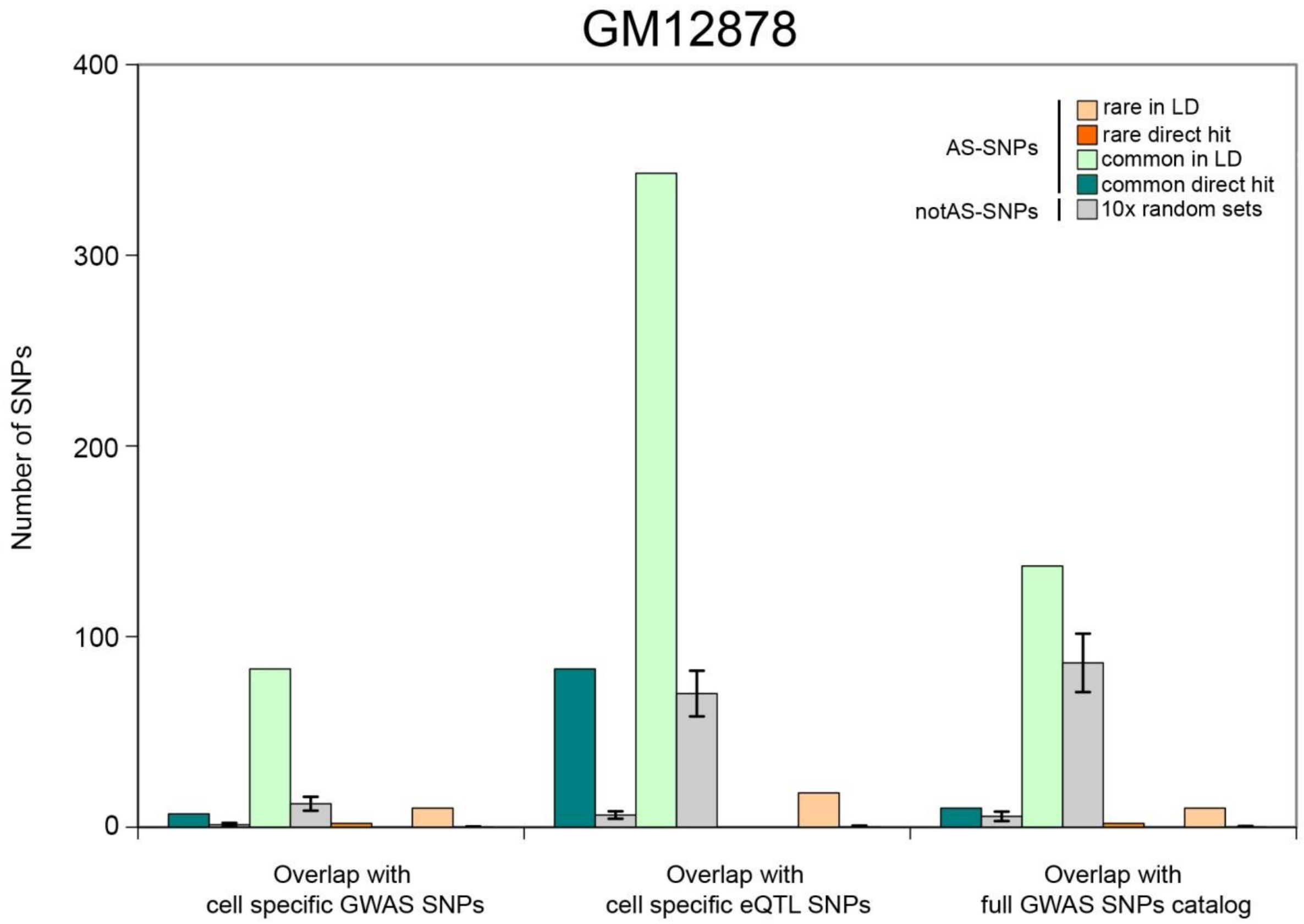

Fig. S3. Overlap of GM12878 AS-SNPs and random sets of non-AS-SNPs with GWAS and eQTL SNPs. Color code as per Fig. 3A. The overlap of each AS-SNPs set based on allele frequency and LD in GM12878 was compared to the average overlap of 10 sets of random selected non-AS-SNPs of the same size (gray bars).

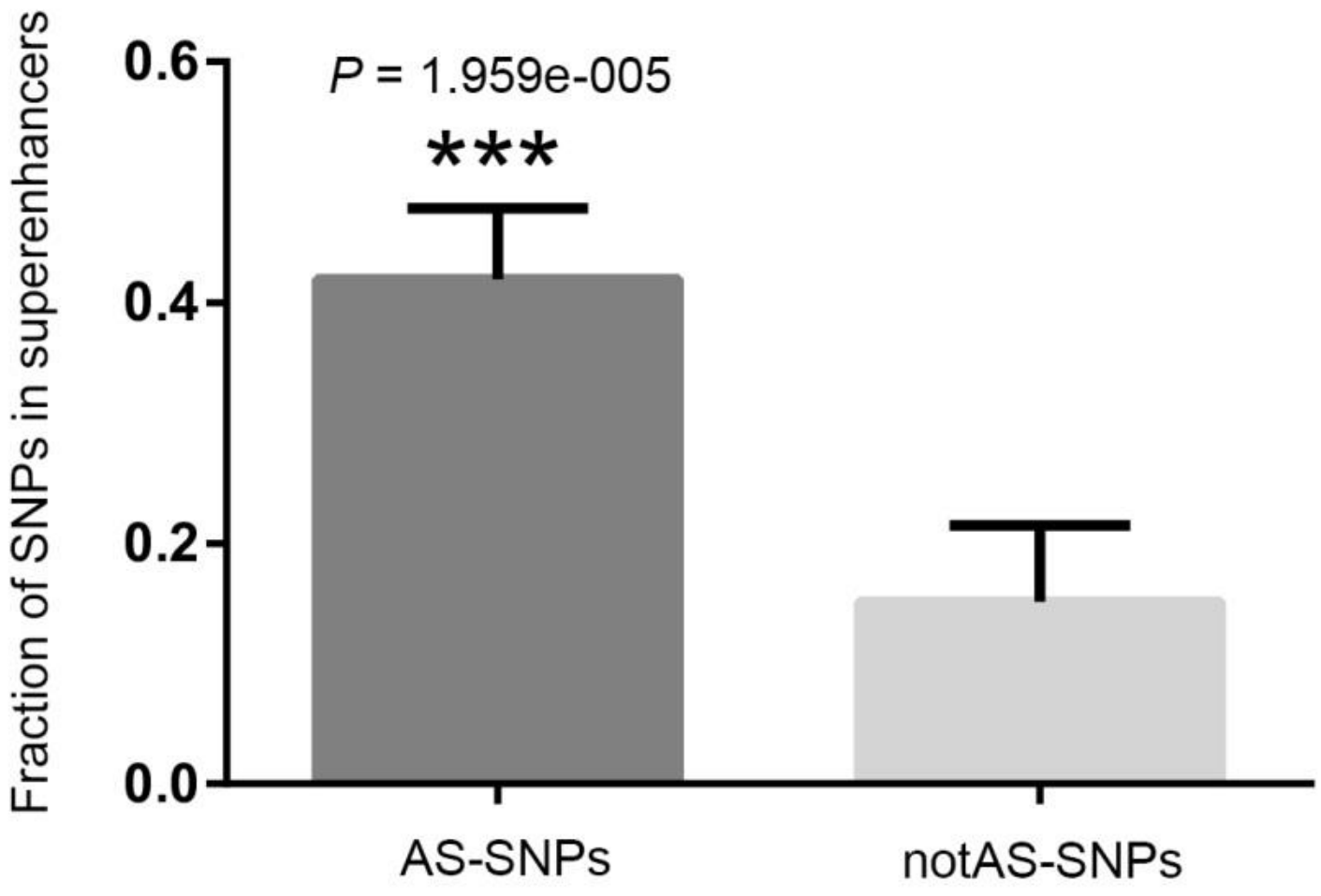

Fig. S4. Overlap of AS-SNPs and a set of random selected non-AS-SNPs with a collection of ~ 60000 super-enhancers (dbSUPER, http://bioinfo.au.tsinghua.edu.cn/dbsuper/index.php). The average fraction of AS-SNPs overlapping super-enhancers in six cell lines is compared to the average overlapping fraction of six random sets of the same size.

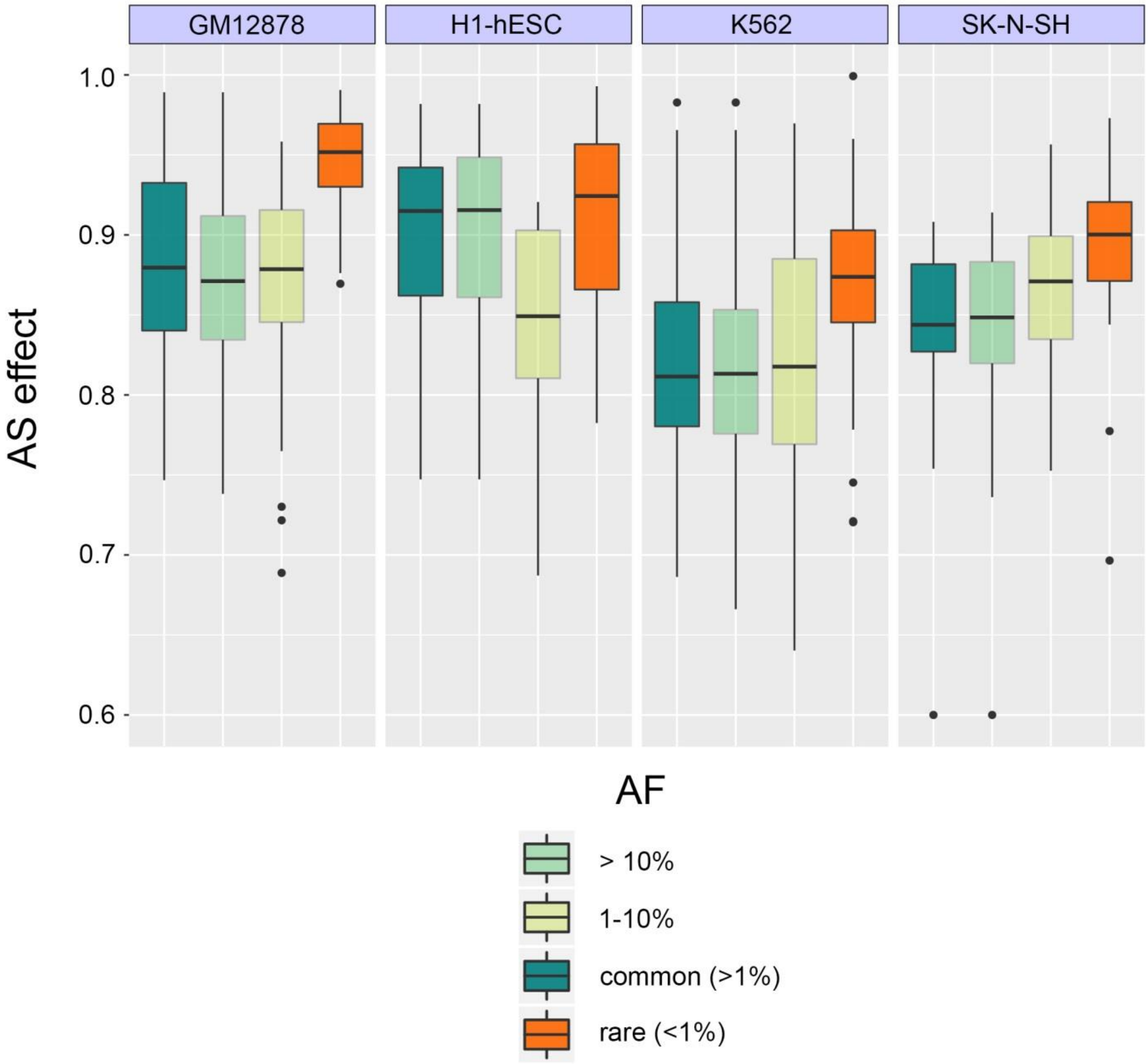

Fig. S5. Allele specific (AS) effect as a function of allele frequency (AF). Boxplot representation of the AS effect shown by AS-SNPs as a function of the allele frequency for four different cell lines. No differences were observed between the common (green gradient boxplots) AF partitions with the exception of H1-hESC 1-10% as a results of few outlier AS-SNPs -defined by TAF7, MAX, NRSF and POL2- showing lower G1/G2 ratio. Color code as per Fig. 1.

Table S1. ChIP-seq reads aligned on reference (G1) and alternative (G2) genomes.

	G1	G2	G1 % of tot	G2 % of tot	
**GM12878**	4,236,153,239	4,236,408,319	50,0	50,0	genome wide
**K562**	5,003,310,513	5,004,308,467	50,0	50,0	
**SK-N-SH**	1,831,058,315	1,832,163,653	50,0	50,0	
**H1-hESC**	2,362,310,577	2,402,228,297	49,6	50,4	
**GM12878**	14,151,740	14,259,434	49,8	50,2	at hz SNPs
**K562**	11,967,525	11,688,904	50,6	49,4	
**SK-N-SH**	7,378,428	7,391,712	50,0	50,0	
**H1-hESC**	6,294,940	6,311,550	49,9	50,1	

Table S2. Heterozygous and AS-SNPs present in one or several cell lines.

Shared between n cell lines	Heterozygous SNPs	%	AS-SNPs	%
1	3,296,857	59.68	9,215	96.28
2	1,580,106	28.60	324	3.39
3	551,933	9.99	29	0.30
4	94,987	1.72	3	0.03
**Total**	5,523,883		9,571	

Table S3. List of cell-specific traits selected form the GWAS catalog.

**B-cells and autoimmune related traits in GWAS catalog**
Alopecia or Alopecia Areata (AA)
Adverse response to chemotherapy (neutropenia/leucopenia) (carboplatin)
Amyotrophic lateral sclerosis
Anklosing spondylitis
Asthma
Atopic dermatitis
Behcet's syndrome
Celiac disease
Chronic lymphocytic leukemia
Chronic obstructive pulmonary disease
Crohn's Disease
Endometriosis
Graves' Disease
Idiopathic pulmonary fibrosis
IgA levels
IgA nephropathy
IgE elvels
IgE grass sensitization
IgG glycosylation
IgG levels
IgM levels
Inflammatory bowel disease
Kawasaki disease (KD)
Lupus- see Systemic lupus erythematosus
Multiple Sclerosis
Myasthenia gravis
Narcolepsy
Neonatal lupus
Neuromyelitis optica (also Devic's disease)
Pemphigus vulgaris
Primary biliary Cirrhosis see Hanot Syndrome
Primary sclerosing cholangitis (PSC)
Psoriasis
Psoriatic arthritis
Restless leg syndrome
Retinopathy
Rheumatoid arthritis (RA)
Sarcoidosis
Schizophrenia
Self-reported allergy
Systemic lupus erythematosus
Systemic sclerosis (scleroderma)
Type 1 diabetes
Ulcerative colitis
Vitiligo

Leukemia related traits in GWAS catalog
Acute lymphoblastic leukemia (B-cell precursor)
Acute lymphoblastic leukemia (childhood)
Acute myeloid leukemia
Adverse response to chemotherapy (neutropenia/leucopenia) (carboplatin)
Asparaginase hypersensitivity in acute lymphoblastic leukemia
Asthma
B cell non-Hodgkin lymphoma
CD4:CD8 lymphocyte ratio
Chronic lymphocytic leukemia
Chronic myeloid leukemia
Follicular lymphoma
Hodgkin's lymphoma
Large B-cell lymphoma
Lymphoma
Methotrexate clearance (acute lymphoblastic leukemia)
Methotrexate phramacokinetics (acute lymphoblastic leukemia)
Myeloproliferative neoplasms
Nodular sclerosis Hodgkin lymphoma
Response to treatment for acute lymphoblastic leukemia
Self-reported allergy
White blood cell count
White blood cell types
**Neuroblastoma and brain related traits in GWAS catalog**
5-HTT brain serotonin transporter levels
Alzheimer's disease (late onset)
Amyotrophic lateral sclerosis
Anorexia nervosa
Attention deficit hyperactivity disorder (time to onset)
Autism
Bipolar disorder
Bipolar disorder (age of onset and psychomotor symptoms)
Bipolar disorder (mania)
Bipolar disorder (mood-incongruent)
Bipolar disorder and major depressive disorder (combined)
Bipolar disorder and schizophrenia
Brain connectivity
Brain cytoarchitecture
Brain imaging in schizophrenia (interaction)
Brain lesion load
Brain structure
Brain structure (hippocampal volume)
Brain structure (temporal lobe volume)
Depression (quantitative trait)
Depression and alcohol dependence
Event-related brain oscillations
Formal thought disorder in schizophrenia
Glioblastoma
Glioma
Glioma (high-grade)
Inattentive symptoms
Migraine

Multiple sclerosis--Brain Glutamate Levels
Neuroblastoma
Neuroblastoma (high-risk)
Normalized brain volume
Obesity-related traits
Parkinson's disease
Schizophrenia
Schizophrenia (cytomegalovirus infection interaction)
Schizophrenia (negative symptoms)
Suicide attempts in bipolar disorder
Temperament (bipolar disorder)
Volumetric brain MRI
White matter integrity

Table S4. Top 20 TFs, polymerases or coactivators detecting most AS-SNPs in 4 cell lines.

GM12878	H1-hESC	SK-N-SH	K562
CTCF	CTCF	CTCF	CTCF
POL2	POL2	POL2	POL2
RAD21	RAD21	RAD21	RAD21
YY1	YY1	YY1	YY1
TBP	TBP	NRSF	TBP
NRSF	NRSF	MAX	USF1
ZNF143	USF1	USF1	MAX
P300	MAX	RFX5	CEBPB
EGR1	CEBPB	GABP	GABP
ELF1	ZNF143	JUND	ELF1
NFYB	JUND	P300	NFYB
CREB1	RFX5	ZBTB33	TEAD4
POU2F2	TEAD4	FOSL2	EGR1
BCL3	CREB1	SMC3	E2F6
PAX5	E2F6	MXI1	HEY1
SRF	MAFK	NRF1	GATA2
BATF	TAF1	NFIC	CJUN
PU1	SIN3	PBX3	BHLHE40
RUNX3	TAF7	TCF12	TAL1
EBF1	NANOG	GATA3	CMYC

Number of cell lines	
(dark red)	x4
(orange)	x3
(yellow)	x2
(lavender)	x1

Table S5. Fraction of heterozygous SNPs in one or more individuals based on allele frequencies.

	p (MAF)	Hz in 1 person	Hz in 2 people	Hz in 3 people	Hz in 4 people	Hz in 5 people	Hz in 10 people
RARE	**0,0010**	0,002	0,004	0,006	0,008	0,010	0,020
	**0,0015**	0,003	0,006	0,009	0,012	0,015	0,030
	**0,0020**	0,004	0,008	0,012	0,016	0,020	0,039
	**0,0025**	0,005	0,010	0,015	0,020	0,025	0,049
	**0,0050**	0,010	0,020	0,030	0,039	0,049	0,095
	**0,0075**	0,015	0,030	0,044	0,058	0,072	0,139
	**0,01**	0,020	0,039	0,058	0,077	0,095	0,181
COMMON	**0,02**	0,039	0,077	0,113	0,148	0,181	0,330
	**0,03**	0,058	0,113	0,165	0,213	0,259	0,451
	**0,04**	0,077	0,148	0,213	0,274	0,329	0,550
	**0,05**	0,095	0,181	0,259	0,329	0,393	0,631
	**0,06**	0,113	0,213	0,302	0,380	0,450	0,698
	**0,07**	0,130	0,243	0,342	0,428	0,502	0,752
	**0,08**	0,147	0,273	0,380	0,471	0,549	0,797
	**0,09**	0,164	0,301	0,415	0,511	0,591	0,833
	**0,10**	0,180	0,328	0,449	0,548	0,629	0,863
	**0,11**	0,196	0,353	0,480	0,582	0,664	0,887
	**0,12**	0,211	0,378	0,509	0,613	0,695	0,907
	**0,13**	0,226	0,401	0,537	0,641	0,723	0,923
	**0,14**	0,241	0,424	0,562	0,668	0,748	0,936
	**0,15**	0,255	0,445	0,587	0,692	0,771	0,947
	**0,16**	0,269	0,465	0,609	0,714	0,791	0,956
	**0,17**	0,282	0,485	0,630	0,735	0,809	0,964
	**0,18**	0,295	0,503	0,650	0,753	0,826	0,970
	**0,19**	0,308	0,521	0,668	0,770	0,841	0,975
	**0,20**	0,320	0,538	0,686	0,786	0,855	0,979
	**0,21**	0,332	0,554	0,702	0,801	0,867	0,982
	**0,22**	0,343	0,569	0,717	0,814	0,878	0,985
	**0,23**	0,354	0,583	0,731	0,826	0,888	0,987
	**0,24**	0,365	0,597	0,744	0,837	0,897	0,989
	**0,25**	0,375	0,609	0,756	0,847	0,905	0,991
	**0,26**	0,385	0,622	0,767	0,857	0,912	0,992
	**0,27**	0,394	0,633	0,778	0,865	0,918	0,993
	**0,28**	0,403	0,644	0,787	0,873	0,924	0,994
	**0,29**	0,412	0,654	0,796	0,880	0,930	0,995
	**0,30**	0,420	0,664	0,805	0,887	0,934	0,996
	**0,31**	0,428	0,673	0,813	0,893	0,939	0,996
	**0,32**	0,435	0,681	0,820	0,898	0,943	0,997
	**0,33**	0,442	0,689	0,826	0,903	0,946	0,997
	**0,34**	0,449	0,696	0,833	0,908	0,949	0,997
	**0,35**	0,455	0,703	0,838	0,912	0,952	0,998
	**0,36**	0,461	0,709	0,843	0,915	0,954	0,998
	**0,37**	0,466	0,715	0,848	0,919	0,957	0,998
	**0,38**	0,471	0,720	0,852	0,922	0,959	0,998
	**0,39**	0,476	0,725	0,856	0,924	0,960	0,998
	**0,40**	0,480	0,730	0,859	0,927	0,962	0,999
	**0,41**	0,484	0,734	0,862	0,929	0,963	0,999
	**0,42**	0,487	0,737	0,865	0,931	0,965	0,999
	**0,43**	0,490	0,740	0,868	0,932	0,966	0,999
	**0,44**	0,493	0,743	0,870	0,934	0,966	0,999
	**0,45**	0,495	0,745	0,871	0,935	0,967	0,999
	**0,46**	0,497	0,747	0,873	0,936	0,968	0,999
	**0,47**	0,498	0,748	0,874	0,937	0,968	0,999
	**0,48**	0,499	0,749	0,874	0,937	0,968	0,999
	**0,49**	0,500	0,750	0,875	0,937	0,969	0,999
	**0,50**	0,500	0,750	0,875	0,938	0,969	0,999

Table S6. Oligonucleotide sequences used in this study.

**RT-qPCR primers**	
EBF1-F	ATCAAAGCCATCAGCCCGAG
EBF1-R	TGACTTCCACAACACCAGGG
SYNGR1-F	TCTGCATCTACAACCGCAAC
SYNGR1-R	GTTGGCCAGGTAGCAGAATC
COG6-F	TTTTTCAAGGCACTGGGAAG
COG6-R	TTGAGCCCATCGGTAAAGTC
ACTB-F	GACCTGTACGCCAACACAGTGC
ACTB-R	CCGATCCACACGGAGTACTTGC
